# BRUTUS at the crossroad of iron uptake and nodulation

**DOI:** 10.1007/s00299-025-03584-w

**Published:** 2025-08-05

**Authors:** Chandan Kumar Gautam, Barney A. Geddes

**Affiliations:** Department of Microbiological Sciences, North Dakota State University, Fargo, ND USA

**Keywords:** Iron signaling, Nodulation, Nitrogen fixation, BTSa, IMA peptides

## Abstract

The functional divergence of GmBTSa in legumes supports iron availability through the activation of NSP–NIN, essential for nodulation.

## Iron’s essential role in nodulation

Iron (Fe) is an essential micronutrient for plants and plays a crucial role in supporting symbiotic events during nodulation. Most of our understanding of Fe uptake and homeostasis comes from research on the Arabidopsis model system, which follows Strategy I (reduction-based) Fe uptake, a mechanism employed by dicots including legumes. While it has long been established that Fe is necessary for nitrogenase activity and successful nitrogen (N) fixation in legumes, only recently have we begun to uncover how Fe regulatory pathways intersect with nodulation signaling. Recent research by Ren et al. (2025) has identified BRUTUS A (BTSa), an Fe-binding E3 ligase as a key molecular hub linking Fe availability to nodulation. This discovery not only reiterates the existence of parallel Fe sensing strategies between Arabidopsis and legumes but also reveals a possible divergence in the BRUTUS function in legumes to cater to the Fe demanding N fixation events.

## Parallels between molecular events during Fe uptake in Arabidopsis and legumes

To adapt to fluctuating environmental Fe levels, plants have evolved intricate regulatory networks to sense Fe status and modulate the expression of Fe uptake and transport genes. In Arabidopsis, Fe deficiency triggers a transcriptional cascade where FE-DEFICIENCY INDUCED TRANSCRIPTION FACTOR1 (FIT1) interacts with Ib-subgroup basic helix–loop–helix (bHLH) transcription factors (TFs) (bHLH38/39/100/101) to activate genes involved in reduction (*FERRIC REDUCTION OXIDASE2, FRO2*) of poorly available oxidized Fe and transport (*IRON-REGULATED TRANSPORTER1*, *IRT1*) of reduced Fe into the roots (Trofimov et al. 2024). This response is further regulated by upstream IVb-subgroup (bHLH121/URI) and IVc-subgroup bHLH TFs (bHLH34, bHLH104, bHLH105/ILR3 and bHLH115). Under Fe-sufficient conditions, BTS/ BTS-LIKE proteins (BTSLs) sense Fe and interact with and ubiquitinate bHLH121/URI, FIT, and IVc bHLH TFs, leading to their degradation and suppression of the Fe deficiency response (Rodríguez-Celma et al. 2019; Trofimov et al. 2024). However, during Fe deficiency, IRONMAN (IMA) peptides are abundantly produced to counteract BTS activity by competing with bHLH105/bHLH115 for interaction, preventing their degradation and sustaining the activation of the Fe-uptake pathway (Li et al. 2021).

In legumes, the regulation of Fe uptake and distribution is complex owing to the presence of N-fixing nodules, specialized root structures with a high demand of Fe. In addition, the potential crosstalk of Fe and nitrate can further influence this regulation. Nodule-specific transporters such as VACUOLAR IRON TRANSPORTERS (VITs), MULTIDRUG AND TOXIC COMPOUND EXTRUSION (MATE), NATURAL RESISTANCE-ASSOCIATED MACROPHAGE PROTEIN1 (NRAMP1), and YELLOW STRIPE-LIKE7 (YSL7), and peptides like NODULE-SPECIFIC CYSTEINE-RICH (NCR247) have been identified as crucial for maintaining Fe levels within the nodules and thus nodulation (Brear et al. 2013; Liu et al. 2023).

At systemic level, the parallel molecular pathways in legumes still remain to be fully characterized. In soybean, the TFs *GmbHLH57* and *GmbHLH300* which are putative homologues of *AtFIT* and *AtbHLH Ib* have been shown to be upregulated in both roots and nodules under Fe deficiency. In addition, like Arabidopsis, they also induce the expression of Fe-responsive genes, *GmFRO2* and *GmIRT1* (Li et al. 2018)*.* Further, GmbHLH300 influences Fe distribution in nodules by negatively regulating *GmYSL7*. It is also involved in transcriptional repression of *EARLY NODULIN93 (GmENOD93)*, a positive regulator of nodule number and nitrogenase activity (Wu et al. 2023).

In *Lotus japonicus*, the *LjIMA1/2* peptides have been recently reported to be upregulated under Fe deficiency and during rhizobia symbiosis. These peptides influence the Fe levels inside the nodules at both local and systemic levels. Interestingly, *LjIMA*s are also induced by N sources even in the absence of rhizobia. Upon the supplementation of Fe, their N-dependent transcription level is reduced, thereby pointing towards the role of IMAs in maintaining Fe–N balance.(Ito et al. 2024).

## Functional divergence between GmBTSa and AtBTS/BTSL

**Fig. 1:**
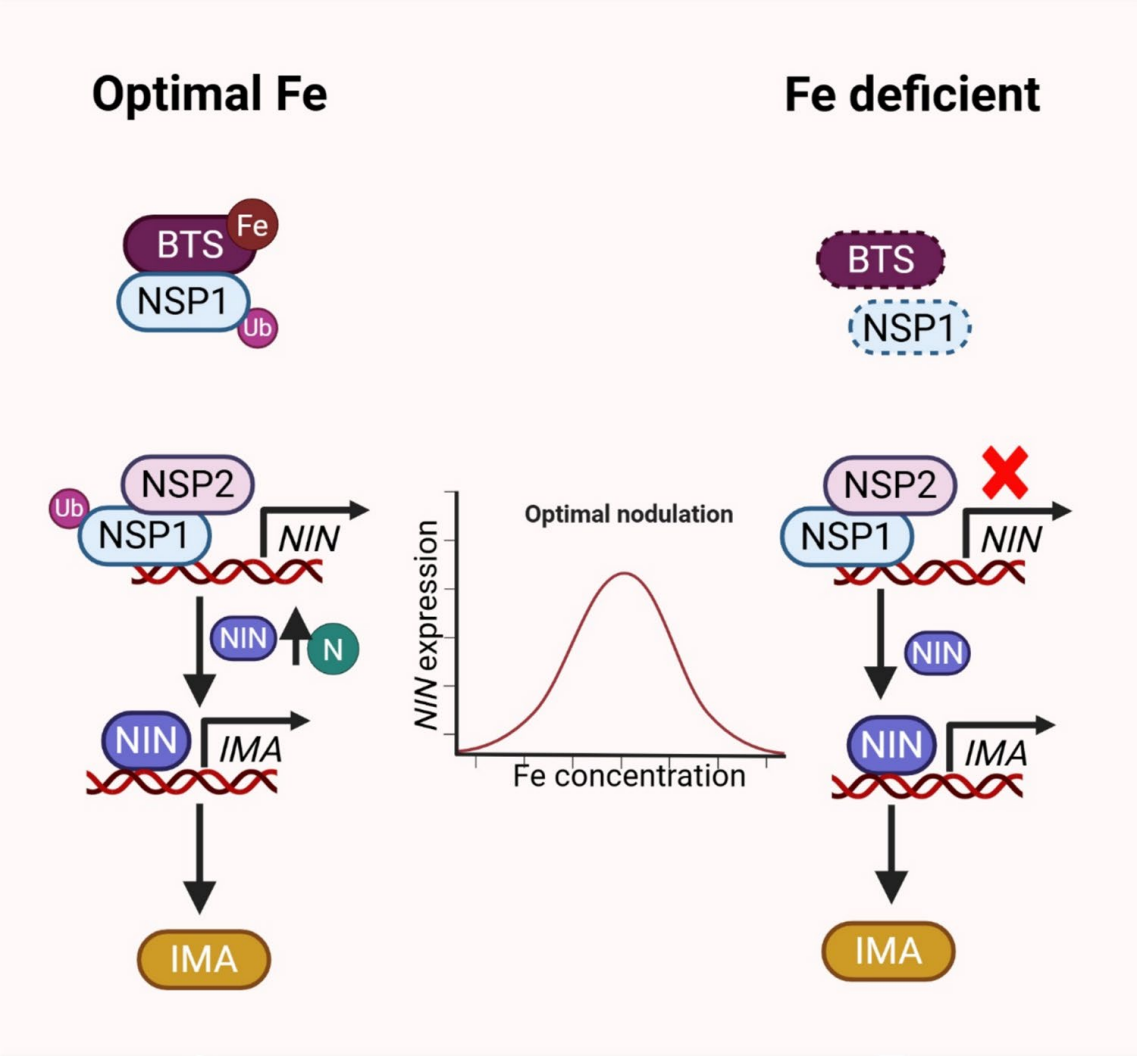
Mode of action of BTS during nodulation under optimal and iron-deficient conditions. Under optimal iron (Fe) conditions in soybean, BRUTUS (BTS) binds Fe and becomes stabilized, which in turn facilitates the monoubiquitination and stabilization of NODULATION SIGNALING PATHWAY1 (NSP1). Stabilized NSP1 regulates the expression of NODULE INCEPTION (NIN), a key nodulation regulator whose expression is Fe-dosedependent and aligns with optimal nodule formation. In contrast, under Fe-deficient conditions, BTS becomes destabilized and is degraded, leading to the degradation of NSP1 and subsequent disruption of downstream nodulation pathways. In another legume, *L. japonicus*, LjNIN regulates the expression of* (IRONMAN, IMA) LjIMA*, which is upregulated under low Fe and high nitrogen (N) conditions. This regulatory relationship makes the potential interaction between IMA and BTS in legumes uncertain, as IMA, which would typically compete with bHLH TFs during Fe deficiency, is itself regulated by NIN. In the accompanying figure, solid lines represent established pathways, and degradation is represented by a dotted box outline, where Ub represents ubiquitination; N, nitrogen; and Fe, iron. Image was created using BioRender.

During nodulation, the perception of Nod factors triggers a cascade of signaling events that activate key GRAS-type TFs, including NODULATION SIGNALING PATHWAY (NSP1 and NSP2), which in turn regulate downstream genes such as *NODULE INCEPTION* (*NIN*). Ren et al. 2025 reported that GmBTSa is upregulated under low Fe conditions and promotes nodulation by stabilizing NSP1 through monoubiquitination, a modification that also enhances NSP1 binding affinity to the NIN1a promoter. This regulatory mechanism appears to be conserved in other legumes as well, such as *L. japonicus* and *Medicago truncatula*, suggesting that NSP1 monoubiquitination by GmBTSa represents a symbiotic adaptation (Fig. 1). Notably, although GmBTSa and its Arabidopsis homologues AtBTS/BTSLs all function as Fe sensors, they exhibit significant differences in their response to Fe availability and regulatory roles. While AtBTS/BTSLs negatively regulate Fe uptake by polyubiquitinating bHLH-type TFs leading to their degradation under Fe-sufficient condition, GmBTSa requires Fe binding for its stability and positively regulates nodulation by monoubiquitinating and stabilizing NSP1, thereby enhancing the expression of downstream symbiotic genes. This Fe-dependent stabilization of GmBTSa likely ensures that nodulation is promoted only when Fe is sufficiently available to support N fixation, highlighting an evolved, legume-specific mechanism that diverges from the known role of BTS proteins in Fe homeostasis.

Furthermore, *GmNINs* whose promoters have binding sites for NSP1 exhibit Fe dose-dependent upregulation in the presence of rhizobia, with their peak expression aligning with the Fe concentration that supports optimal nodulation efficiency, as reflected by the highest nodule numbers, infection thread formation, and nitrogenase activity (Ren et al. 2025). In *L. japonicus*, LjNIN positively regulates the expression of *LjIMA1*, a peptide responsive to Fe levels, through direct interaction at the NIN-binding sequence (NBS) and the nitrate-responsive cis-element (NRE) within the *LjIMA1* promoter (Ito et al. 2024). Assuming this regulatory mechanism is conserved across legumes, a plausible explanation for the differing stability of GmBTSa in the presence of Fe could lie in the regulatory divergence between legumes and Arabidopsis. While in Arabidopsis, *IMAs* respond directly to Fe levels, in legumes, their transcription is mediated by *NIN* whose expression is tightly restricted to a narrow range of Fe availability. Nevertheless, *LjIMA1* and *LjIMA2* behave similar to their Arabidopsis counterparts, being upregulated under both Fe deficiency and high N conditions (Ito et al. 2024). Overexpression of *IMAs* led to the induction of several key Fe-responsive genes in both *L. japonicus* and Arabidopsis, including *FRO2* and *IRT1*, which are not strongly associated with root nodule symbiosis, reflecting a conserved function of downstream players (Grillet et al. 2018; Ito et al. 2024).

## Conclusions

The coordination of Fe sensing and nodulation signaling through GmBTSa, coupled with Fe-dependent regulation of *GmNINs*, reveals an intricate regulatory network that synchronizes nutrient availability and symbiotic efficiency in legumes (Fig. 1). The alignment of peak *GmNINs* expression with optimal nodulation suggests a precisely coordinated mechanism to maximize N fixation under optimal Fe conditions. The functional parallels observed among legumes suggest that similar to LjNIN regulation of *LjIMA*, the GmNIN-*GmIMA* interaction may also be involved in fine-tuning Fe homeostasis to enhance plant fitness during symbiosis. A critical unresolved question is whether legume IMAs interact with BTS homologues (as observed in Arabidopsis) or compete with NSP1 to balance Fe uptake and nodulation. In Arabidopsis, BTS acts paradoxically as a negative regulator of Fe acquisition genes despite its upregulation under Fe deficiency, destabilizing bHLH transcription factors to prevent toxic overaccumulation. This homeostatic feedback contrasts with the legume system, where GmBTSa and GmNIN likely co-opt similar components to prioritize symbiotic demands over generic Fe homeostasis. Future studies must address whether legume IMAs compete with bHLH TFs or NSP1 and how this interplay adapts rhizobial symbiosis to fluctuating Fe environments.
