# Correction: BRUTUS at the crossroad of iron uptake and nodulation

**DOI:** 10.1007/s00299-025-03646-z

**Published:** 2025-11-24

**Authors:** Chandan Kumar Gautam, Barney A. Geddes

**Affiliations:** Department of Microbiological Sciences, North Dakota State University, Fargo, ND USA

**Correction: Plant Cell Reports** 10.1007/s00299-025-03584-w

A portion of caption of figure 1 was inadvertently placed as main text before conclusion part.

The complete correct Fig. 1 and caption are given below.

**Fig. 1:**
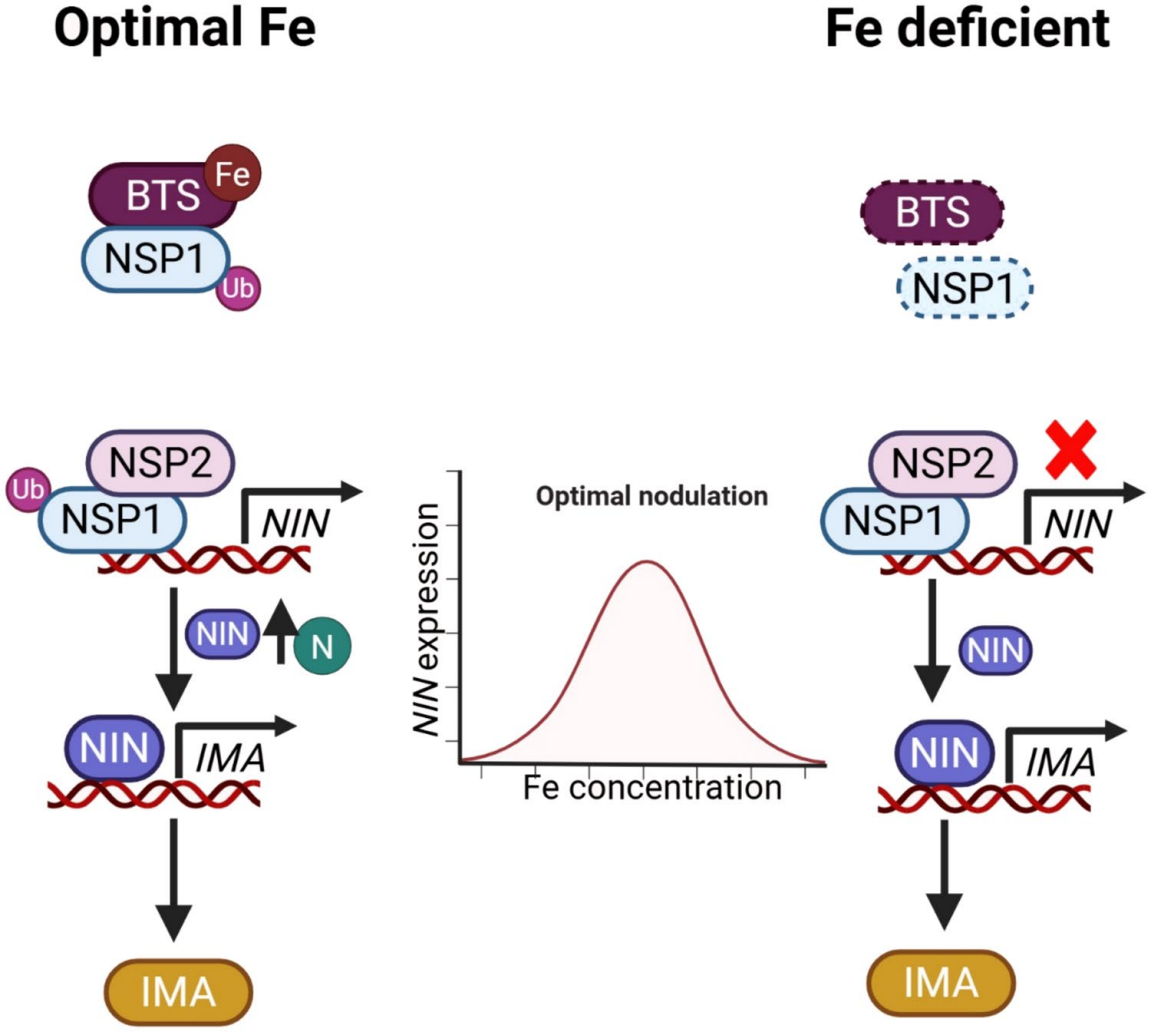
Mode of action of BTS during nodulation under optimal and iron-deficient conditions. Under optimal iron (Fe) conditions in soybean, BRUTUS (BTS) binds Fe and becomes stabilized, which in turn facilitates the monoubiquitination and stabilization of NODULATION SIGNALING PATHWAY1 (NSP1). Stabilized NSP1 regulates the expression of NODULE INCEPTION (NIN), a key nodulation regulator whose expression is Fe-dose-dependent and aligns with optimal nodule formation. In contrast, under Fe-deficient conditions, BTS becomes destabilized and is degraded, leading to the degradation of NSP1 and subsequent disruption of downstream nodulation pathways. In another legume, *L. japonicus*, LjNIN regulates the expression of *(IRONMAN, IMA) LjIMA*, which is upregulated under low Fe and high nitrogen (N) conditions. This regulatory relationship makes the potential interaction between IMA and BTS in legumes uncertain, as IMA, which would typically compete with bHLH TFs during Fe deficiency, is itself regulated by NIN. In the accompanying figure, solid lines represent established pathways, and degradation is represented by a dotted box outline. Ub represents ubiquitination; N, nitrogen; and Fe, iron. Image was created using BioRender

The original article has been corrected.

This error was introduced during the publication process from Springer service provider.

Apart from this everything remains unchanged and correct.

